# Enabling multiplexed testing of pooled donor cells through whole-genome sequencing

**DOI:** 10.1186/s13073-018-0541-6

**Published:** 2018-04-19

**Authors:** Yingleong Chan, Ying Kai Chan, Daniel B. Goodman, Xiaoge Guo, Alejandro Chavez, Elaine T. Lim, George M. Church

**Affiliations:** 1 Wyss Institute for Biologically Inspired Engineering, Harvard University, Boston, MA 02115 USA; 2 Department of Genetics, Harvard Medical School, Boston, MA 02115 USA; 3 Harvard-MIT Health Sciences and Technology, Cambridge, MA 02139 USA; 4 Department of Pathology and Cell Biology, Columbia University College of Physicians and Surgeons, New York, NY 10032 USA

**Keywords:** Multiplexed testing, Barcode free method, Single nucleotide polymorphisms, Expectation maximization algorithm, Next-generation sequencing, Personal Genome Project

## Abstract

We describe a method that enables the multiplex screening of a pool of many different donor cell lines. Our method accurately predicts each donor proportion from the pool without requiring the use of unique DNA barcodes as markers of donor identity. Instead, we take advantage of common single nucleotide polymorphisms, whole-genome sequencing, and an algorithm to calculate the proportions from the sequencing data. By testing using simulated and real data, we showed that our method robustly predicts the individual proportions from a mixed-pool of numerous donors, thus enabling the multiplexed testing of diverse donor cells *en masse*.

More information is available at https://pgpresearch.med.harvard.edu/poolseq/

**Electronic supplementary material:**

The online version of this article (10.1186/s13073-018-0541-6) contains supplementary material, which is available to authorized users.

## Background

The screening of many cell lines for specific phenotypes is commonly performed to discover factors that confer donor cell specific effects. For example, several studies have employed the screening of multiple cancer cell lines for identifying cell type specific essential genes [1–3]. Other studies have also used primary cells from different donors to identify genetic variants associated with various cellular phenotypes. In one study, the authors reported six loci associated with immune response to pathogens by measuring cytokine production in peripheral blood mononuclear cells from hundreds of different donors [4]. Other groups measured the transcriptional response to pathogenic stimulus in primary monocytes obtained from many African and European individuals [5, 6]. In these studies, the experiments were performed on cells from each individual donor separately. However, with increasing numbers of donors, generating data from cells from more donors would require more research effort and time. As such, it would be advantageous to multiplex these assays by performing a single experiment on a pool of all donor cells and simultaneously retrieve phenotypic data from each donor.

**Fig. 1:**
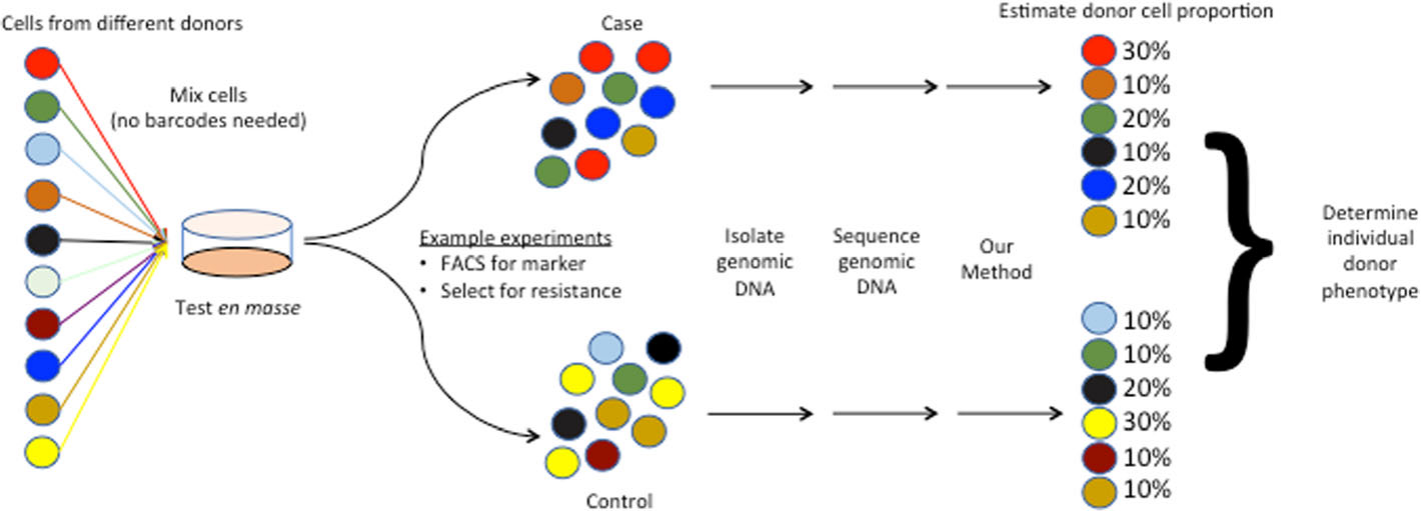
Workflow of how our method is used for testing cells from multiple donors *en masse*. Using FACS or selection, one can obtain the case and control group of cells. The individual donor proportions for the case and control group can be obtained using our method and thus each individual donor can be assigned a phenotype value. The method does not require artificial barcodes or amplification of a specific locus

To achieve this, one would require a method to accurately estimate the individual proportion of each donor from a pool of cells containing multiple donors. With such a method, one can perform a selection assay or perform fluorescence-activated cell sorting (FACS) to sort the pool of cells based on criteria of interest (e.g. response to pathogen, drug resistance, protein expression) and identify the proportion of every individual donor within this new pool (case group). A similar experiment can be performed for the control group, to identify the donor proportion either at baseline or from cells sorted with different criteria. The phenotype for an individual donor is then measured by comparing the difference in proportion between case and control groups (Fig. 1). A recent study aimed at discovering genotype-specific effects in a mixture of cancer cells reported a method (PRISM) that achieved this [7]. Briefly, PRISM uses a unique 24-nt barcode that was integrated into each donor cell line by lentiviral delivery before pooling. To obtain individual donor proportions, the barcodes were amplified using polymerase chain reaction (PCR) and sequenced by next-generation sequencing. Each individual donor proportion is then estimated by calculating the proportion of their corresponding barcodes from the sequenced reads. However, the PRISM method requires the barcoding of individual donor cells using lentiviral delivery, which is a tedious process because each lentiviral barcode has to be generated, applied to the donor cells, and selected for separately. Furthermore, primary cells, non-dividing cells, and cells with limited ability to be passaged in vitro cannot be effectively barcoded in this manner. Here, we describe a method that can accurately estimate each donor proportion in a mixed pool without the use of exogenous barcodes or amplification of a specific locus using PCR.

Our method harnesses the presence of millions of common single nucleotide polymorphisms (SNPs) within the human genome. These SNPs, which are usually bi-allelic, can be exploited as a natural barcode and are distributed throughout the entire genome. These SNPs are spaced relatively far apart, with approximately one common SNP for every 1000 base pairs in the human genome [8]. The genotypes of these SNPs for each donor are pre-determined before executing the method. These SNP genotypes can be easily acquired using whole-genome genotyping arrays or by performing whole-genome sequencing for each donor. While each individual SNP is not unique, the combination of SNPs throughout the genome is unique to each donor. However, PCR amplification and sequencing of any genomic locus is not adequate enough to cover enough SNPs to uniquely identify an individual donor. As such, our method overcomes this problem by using all the SNPs distributed throughout the host genome. Using the standard process of sequencing a human genome from a library of short DNA fragments, many of the short sequencing reads (200–300 bp) generated will cover a SNP in the human population [9]. Our method works by first extracting genomic DNA from the mixed pool of cells and sequencing it. The method then employs an expectation–maximization (EM) algorithm that takes the genotypes for all the donors as well as the sequencing reads from the mixed pool as input to calculate the individual donor proportion. Using an iterative process, the algorithm determines the donor proportion that best matches the expected allelic fraction with the observed allelic fraction for all the SNPs analyzed.

In this study, we demonstrated the feasibility of our approach by designing simulation experiments to determine how well our method can accurately predict donor proportion. From simulation experiments, we tested a number of scenarios by varying the number of donors, number of SNPs as well as the sequencing read-depth per SNP. We found that in most cases, our method accurately predicts the donor proportion even at the lowest possible read-depth (1X) as long as a sufficient number of SNPs were analyzed (> 500,000 SNPs). Finally, we empirically tested our method by sequencing a mixed pool of human donor cells and demonstrate that our approach can accurately predict donor proportion within the mixed population.

## Methods

### EM algorithm for estimating proportion of individual donors within the pool

We first define θ as the probability or proportion of any individual donor, which is the probability that we are trying to estimate, i.e.

$$ {\displaystyle \begin{array}{c}\theta =\left({P}_1,{P}_2,{P}_3,\dots, {P}_N\right)\\ {}{\theta}_n={P}_n\end{array}} $$

where *P*_*n*_ is the probability or proportion of donor *n* within the pool of *N* donors, the sum of which is 1.

Next, we assume that we only analyze sequenced reads from autosomes and only at SNP positions that are known to be bi-allelic, i.e. having only two alleles, Reference (R) or Alternate (A), although the algorithm can be amended to consider X and/or Y chromosomes as well as also incorporating multiallelic polymorphisms. Given this, we define *Reads* as the number of sequence reads (read-depth) for each allele for each SNP, i.e.

$$ {Reads}_{m,R}= No. of\ observed\ reads\ with\ allele\ R\  at\  SNP\  position\ m $$

$$ {Reads}_{m,A}= No. of\ observed\ reads\ with\ allele\ A\  at\  SNP\  position\ m $$

where *m* is the index defining the SNP at that position.

Next, we assume that the genotypes for all bi-allelic SNPs analyzed for every donor is accurately known. As such, the genotype for each donor for each SNP can only be one of the following states: *RR*, *RA*, or *AA*, i.e.

*SNP*_*m*, *n*_= genotype of donor *n* at SNP *m (RR*, *RA*, or *AA)*.

To estimate θ, we employ an EM algorithm and initialized the values of θ so that each donor has the same starting proportion or probability [10], i.e.

$$ {\uptheta}_n^0=\raisebox{1ex}{$1$}\!\left/ \!\raisebox{-1ex}{$N$}\right. $$

where ${\uptheta}_n^0$ is the proportion or probability estimate of individual *n* at iteration *0*.

Next, we calculate the *Total* function for each SNP given θ, which is the expected number of *R* and *A* alleles given the current estimate of θ, i.e.

$$ {Total}_{m,R}=\sum \limits_{n=1}^N\left\{\begin{array}{c}\kern0.75em {\theta}_n^t\kern4.25em if\ {SNP}_{m,n}= RR\kern1em \\ {}0.5\ast {\theta}_n^t\kern1.75em if\ {SNP}_{m,n}= RA\ \\ {}0\kern4.75em if\ {SNP}_{m,n}= AA\ \end{array}\right. $$

$$ {Total}_{m,A}=\sum \limits_{n=1}^N\left\{\begin{array}{c}\kern0.75em 0\kern4.5em if\ {SNP}_{m,n}= RR\kern0.75em \\ {}0.5\ast {\theta}_n^t\kern1.5em if\ {SNP}_{m,n}= RA\\ {}{\theta}_n^t\kern4em if\ {SNP}_{m,n}= AA\ \end{array}\right. $$

where *m* is the index for each SNP, *R* and *A* represent the respective alleles, and ${\theta}_n^t$ represents the current estimate of θ for individual *n* at the current iteration *t*.

Next, we calculate the likelihood function *L* for each individual given the current estimate of θ by going through all the SNPs (*M* being the total number of SNPs), i.e.

$$ {L}_n=\sum \limits_{m=1}^M{\displaystyle \begin{array}{c}\kern1.5em \frac{\theta_n^t}{Total_{m,R}}\ast {Reads}_{m,R}\kern13.75em if\ {SNP}_{m,n}= RR\kern1em \\ {}0.5\left(\frac{\theta_n^t}{Total_{m,R}}\ast {Reads}_{m,R}+\frac{\theta_n^t}{Total_{m,A}}\ast {Reads}_{m,A}\right)\  if\ {SNP}_{m,n}= RA\ \\ {}\frac{\theta_n^t}{Total_{m,A}}\ast {Reads}_{m,A}\kern13.75em if\ {SNP}_{m,n}= AA\ \end{array}} $$

Finally, we re-estimate θ for each donor for the next iteration, i.e.

$$ {\theta}_n^{t+1}=\frac{L_n}{\sum \limits_{n=1}^N{L}_n} $$

This procedure is repeated until θ converges to a stable estimate, *t* = 2000. The final value of *t* can be adjusted depending on the number of donors and SNPs analyzed. For a sample size of ten donors, we used *t* = 500 as the last iteration. To help explain the algorithm, we provide a working example of estimating the proportion of a mixed pool of five donors (Additional file 1: Note S1). We also included a short description of how our method would be used in a real experimental setting by comparing our method against the lentiviral barcoding method (PRISM) used in Yu et al. [7] (Additional file 1: Note S2).

### Simulating individual donors in a mixed pool and estimating their proportions using the EM algorithm

Individuals were simulated by first defining the value of several variables, namely,

1. *N*, the total number of individual donors;
2. *M*, the total number of SNPs;
3. *X*, the read-depth (coverage) for every SNP.

First, a total of *M* SNPs were simulated by randomly assigning a minor allele frequency (MAF) by drawing from a uniform distribution in the range of 5–50%.

$$ {MAF}_m= random\ number\ between\ 5\% and\ 50\% $$

Next, genotypes for each SNP were randomly assigned according to their MAF to each of the *N* donors, i.e. for any donor at any SNP with a MAF of *f*, the probability of having a genotype of *RR*, *RA*, and *AA* is *f*^*2*^, *2f(1-f)*, and *(1-f)*^*2*^, respectively.

Next, each individual was randomly assigned a copy-number count (*Donor*_*n*_) by drawing from a uniform distribution in the range of 1–10,000 to represent the true number of copies of that donor.

$$ {Donor}_n= random\ number\ between\ 1\  and\ \mathrm{10,000} $$

The true proportion for each donor (*θ*_*n*_) was then calculated by taking their copy-number count divided by the sum of all the copy-number for all donors.

$$ {\theta}_n=\frac{Dono{r}_n}{\sum \limits_{n=1}^N{ Dono r}_n} $$

The sequencing-reads were then simulated by randomly drawing *X* number of alleles from a binomial distribution where the probability of drawing the *R* allele for that SNP (*P*_*m,R*_) is the sum of the true proportion multiplied by the likelihood for drawing the *R* allele given the genotype for that individual, i.e.

$$ {P}_{m,R}=\sum \limits_{n=1}^N\left\{\begin{array}{c}\kern1.00em {\theta}_n\kern4em if\ {SNP}_{m,n}= RR\kern1em \\ {}0.5\ast {\theta}_n\kern0.75em if\ {SNP}_{m,n}= RA\ \\ {}0\kern4.25em if\ {SNP}_{m,n}= AA\ \end{array}\right. $$

The simulation can also be done with regards to the *A* allele by changing the above equation or subtracting from 1 the probability of drawing the *R* allele.

$$ {P}_{m,A}=1-{P}_{m,R} $$

Nonetheless, if the random draw for the read fails to draw the *R* allele, it will be assigned the *A* allele and vice versa. The simulated alleles and SNP genotypes for all *N* individuals are then used as inputs to the EM algorithm to estimate the individual donor proportion. The estimated proportion is then compared to the true proportion and the accuracy of the prediction is evaluated using the Pearson correlation coefficient (represented as *R*).

### Pooling B-lymphocytes from Personal Genome Project samples

B-lymphocytes from the Harvard Personal Genome Project (PGP) were obtained from the NIGMS Human Genetic Cell Repository at the Coriell Institute for Medical Research (https://www.coriell.org). To create the initial pool of donor cells, we used five distinct pools of B-lymphocytes previously mixed together at approximately equal numbers (Invitrogen Countess) and kept cryopreserved in liquid nitrogen. The five pools of frozen cells were resuscitated and grown overnight separately in upright T25 flasks in a standard incubator at 37 °C with 15 mL of growth media upright (Thermofisher, RPMI 1640 Medium, GlutaMAX™ Supplement, HEPES + 10% fetal bovine serum + 1% Penicillin-Streptomycin [10,000 U/mL]). The pools of cells were counted (Invitrogen Countess) and cells were taken from Pool 1, Pool 2, Pool 3, Pool 4, and Pool 5 at increments of 100,000 cells, i.e. 100,000 cells were taken from Pool 1, 200,000 cells were taken from Pool 2, … and 500,000 cells were taken from Pool 5. The cells were mixed together to form the final pool. To create the subsequent (more accurate) pool of donor cells, a different set of 50 donor cells were resuscitated and cultured for five days separately in 24-well plates in a standard incubator at 37 °C with 0.5 mL of growth media (Thermofisher, RPMI 1640 Medium, GlutaMAX™ Supplement, HEPES + 10% fetal bovine serum + 1% Penicillin-Streptomycin [10,000 U/mL]). On the day of cell sorting, each donor cell was collected in 1.5-mL micro-centrifuge tubes and re-suspended in 0.5 mL of Dulbecco’s Phosphate Buffered Saline (DPBS) solution. The donor cells were then sorted into a single 15-mL conical centrifuge tube containing 5 mL of DPBS (Sony SH800S Cell Sorter). Ten different donors were selected for each of the five pools and 10,000, 20,000, 30,000, 40,000, and 50,000 events were used to sort donors representing pools 1, 2, 3, 4, and 5, respectively.

### DNA extraction, library preparation, and sequencing

Genomic DNA of the initial pool was extracted using the QIAamp DNA FFPE Tissue Kit (QIAGEN). Genomic DNA of the subsequent pool was extracted using the AccuPrep Genomic DNA extraction kit (BioNEER). The extracted genomic DNA of both pools were submitted to Biopolymers facility at Harvard Medical School (https://genome.med.harvard.edu/) for genomic DNA library preparation (Genomic-Seq Wafergen) and subsequent next-generation sequencing using Illumina MiSeq. The DNA from the initial pool resulted in 5,112,179 paired sequencing reads while the DNA from the subsequent pool resulted in 13,111,543 paired sequencing reads that mapped to the human genome. The reads were aligned to the human genome reference sequence (GRCh37/hg19) using bwa (version 0.7.8-r566) [11].

### SNP identification

Whole-genome sequencing information was available for all 102 PGP samples (Complete Genomics) and the genotypes of all bi-allelic SNPs within the autosomes were recorded. We compared the sequencing reads with the recorded SNPs to determine the allele for each SNP sequenced. The final alignment of the sequencing reads for the initial pool resulted in the sequencing of 1,425,723 SNPs at 1.16X coverage while the subsequent pool resulted in the sequencing of 1,988,295 SNPs at 1.23X coverage.

## Results

### An algorithm that accurately predicts the proportion of individuals within a simulated mixed pool

To test the efficacy of our algorithm, we designed and implemented a simulation program to generate simulated data for testing the robustness of the prediction given the number of donors, number of SNPs as well as sequencing read-depth. Taking these parameters as input, the program first randomly simulates the true proportion for each donor within the mixed pool. Next, it generates genotypes for all SNPs and donors by simulating SNPs with MAF randomly selected in the range of 5–50%. Finally, for each SNP, it stochastically samples the number of each of the alleles under a probabilistic model that reflects the true donor proportion according to the assigned read-depth. The program then applies our algorithm on the simulated data to determine how accurately it can predict the individual donor proportion (see “Methods”).

**Fig. 2:**
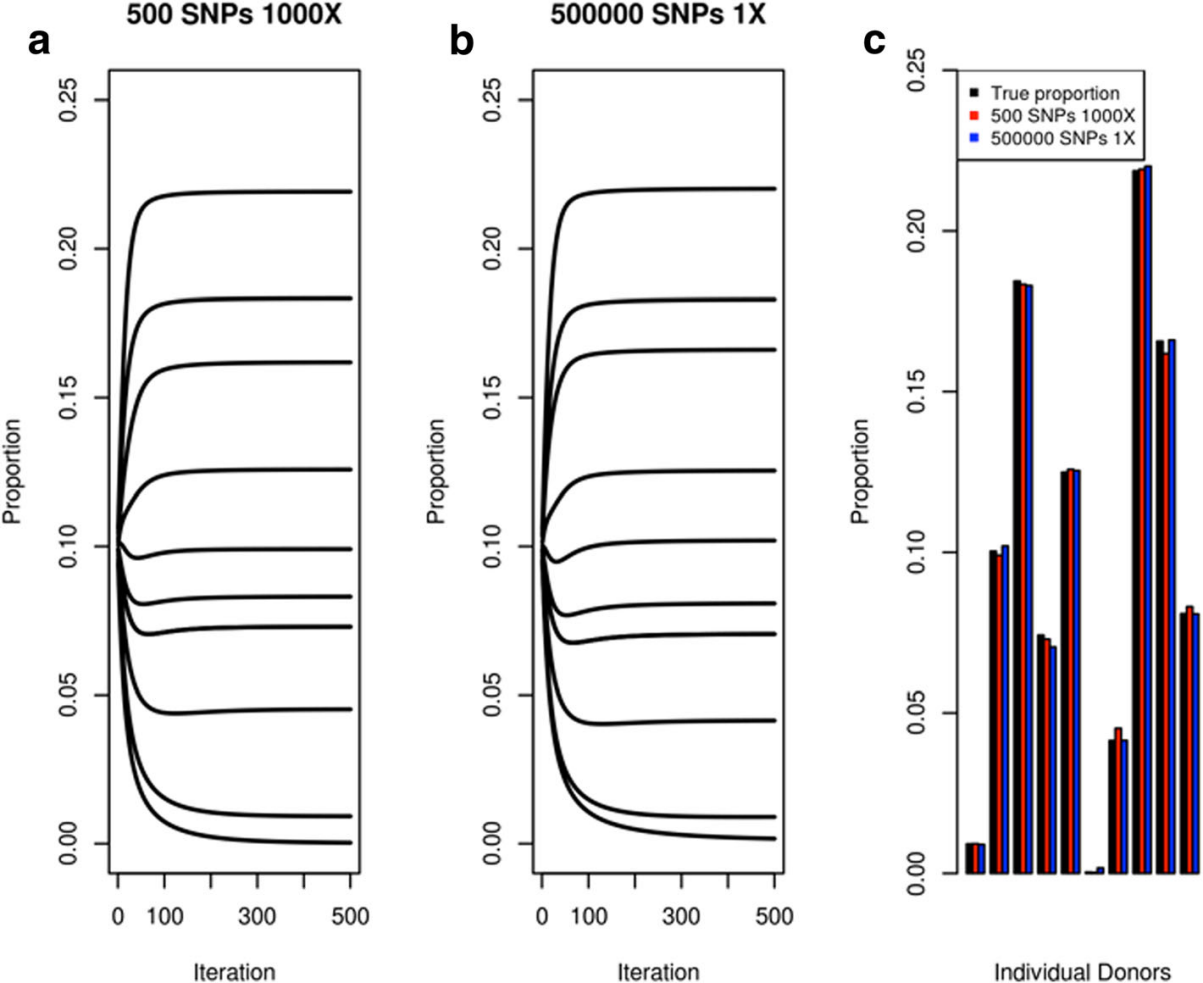
Estimating the proportions of ten simulated donor individuals. Showing the results of simulating (**a**) deep-coverage sequencing (1000X) on a small number (500) of SNPs and (**b**) low-coverage sequencing (1X) on many (500,000) SNPs. Both graph shows the estimated proportion (*y-axis*) by the algorithm at every iteration (*x-axis*). **c** *Bar plot* comparing the estimated proportion against the true proportion for both set A and set B after 500 iterations. The *black bars* represent the true proportion for each simulated donor, while the *red* and *blue bars* represent the estimated proportion of set A and set B, respectively

Using our program, we first simulated two sets of ten diploid individuals with similar proportions, the first (set A) having genotypes from 500 SNPs with sequencing read-depth (coverage) of 1000X while the second (set B) having genotypes from 500,000 SNPs but with sequencing read-depth of only 1X (Additional file 2: Table S1). We ran the algorithm to estimate the individual proportions given the simulated sequencing reads and genotypes of the individuals for both sets and found that the prediction converges to a fixed estimate (Fig. 2a, b) and accurately predicted the real simulated proportion for both set A and set B (Fig. 2c**,** Additional file 2: Table S2). This result shows that the algorithm is as effective on high coverage sequencing data across a small number of SNPs compared with low-coverage sequencing data across a much larger number of SNPs.

### Testing the algorithm on simulated mixed pools by varying the sample size, number of SNPs, and sequencing read-depth

**Fig. 3:**
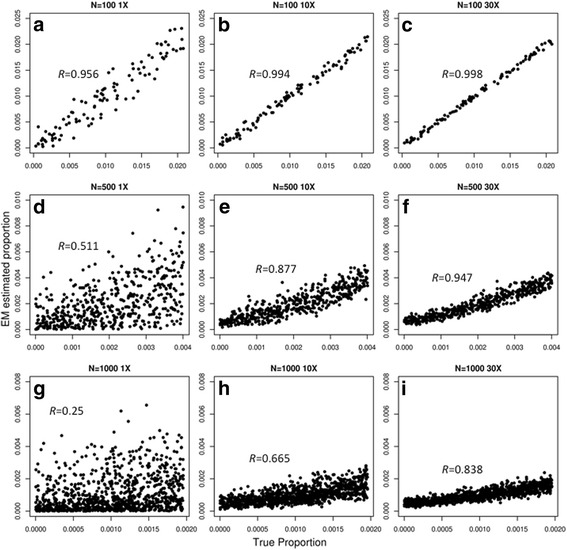
Comparing the true proportions with the estimated proportions of varying number of simulated donor individuals by simulating 500,000 SNPs at varying coverage. The *x-axis* represents the true simulated proportion while the *y-axis* represents the estimated proportion by our algorithm (EM estimated proportion). **a** 100 donors at 1X coverage. **b** 100 donors at 10X coverage. **c** 100 donors at 30X coverage. **d** 500 donors at 1X coverage. **e** 500 donors at 10X coverage. **f** 500 donors at 30X coverage. **g** 1000 donors at 1X coverage. **h** 1000 donors at 10X coverage. **i** 1000 donors at 30X coverage. *R* represents the Pearson-correlation coefficient of comparing the true proportions with the estimated proportions

To test how the number of SNPs and read-depth (coverage) would scale with increased sample size, we perform simulations on pools of 100, 500, and 1000 different donors, using 500,000 SNPs with 1X, 10X, and 30X coverage. For a pool of 100 donors, we obtained Pearson correlation coefficients of 0.956, 0.994, and 0.998 for 1X, 10X, and 30X coverage respectively, demonstrating that under these circumstances, low-coverage sequencing data would be sufficient to accurately predict individual donor proportion (Fig. 3a–c, Additional file 2: Table S3). With a pool of 500 donors, the algorithm produced Pearson correlation coefficients of 0.511, 0.877, and 0.947 for 1X, 10X, and 30X coverage, respectively, indicating a drop in prediction accuracy with increased sample size (Fig. 3d–f). Finally, when the number of donors was increased to 1000, the accuracy further declined for 1X, 10X, and 30X coverage (*R* = 0.25, 0.665, and 0.838, respectively) (Fig. 3g–i). These results show that by analyzing 500,000 SNPs positions, the algorithm can accurately estimate pools of 100 different donors at any read-depth but higher read-depths would be required to accurately estimate donor proportion for pools with substantially more donors.

**Fig. 4:**
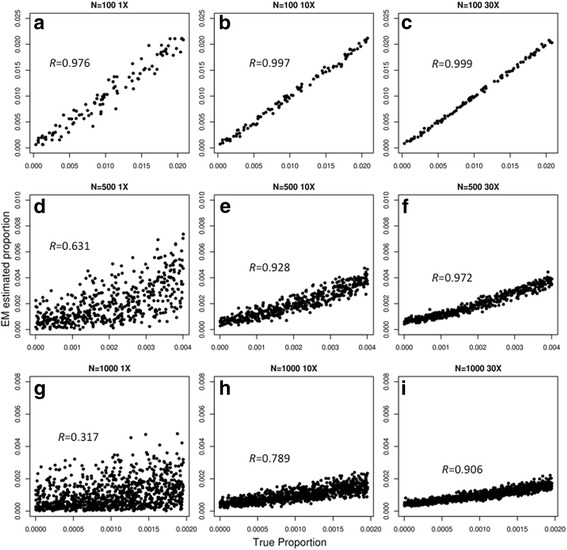
Comparing the true proportions with the estimated proportions of varying number of simulated donor individuals by simulating 1,000,000 SNPs at varying coverage. The *x-axis* represents the true simulated proportion while the *y-axis* represents the estimated proportion by our algorithm (EM estimated proportion). **a** 100 donors at 1X coverage. **b** 100 donors at 10X coverage. **c** 100 donors at 30X coverage. **d** 500 donors at 1X coverage. **e** 500 donors at 10X coverage. **f** 500 donors at 30X coverage. **g** 1000 donors at 1X coverage. **h** 1000 donors at 10X coverage. **i** 1000 donors at 30X coverage. *R* represents the Pearson-correlation coefficient of comparing the true proportions with the estimated proportions

To determine if the accuracy of the algorithm increases with the use of more SNPs in the analysis, we repeated the simulation experiments using 1,000,000 SNPs. Indeed, when we doubled the number of SNPs, the accuracy for all the simulation experiments increased when compared to their previous counterpart (Fig. 4, Additional file 2: Table S4). This suggests that even for a pool of > 100 donors, sequencing more SNPs in general increases the accuracy of the prediction. Based on these results, we tabulated the minimal read-depth required to obtain an accurate prediction with Pearson correlation coefficient ≥ 0.9 (Table 1).

**Table 1:** Minimal read-depth required for accurate prediction of donor proportion

	500,000 SNPs	1,000,000 SNPs
100 donors	1X	1X
500 donors	30X	10X
1000 donors	>30X	30X

The read-depth necessary to obtain an accurate prediction of donor proportion with Pearson correlation coefficient > 0.9 for a mix pool of 100, 500, and 1000 unique donors when 500,000 and 1,000,000 SNPs are analyzed with our method

### The method accurately predicts the donor proportions of a mixed pool of actual human donor cells

**Fig. 5:**
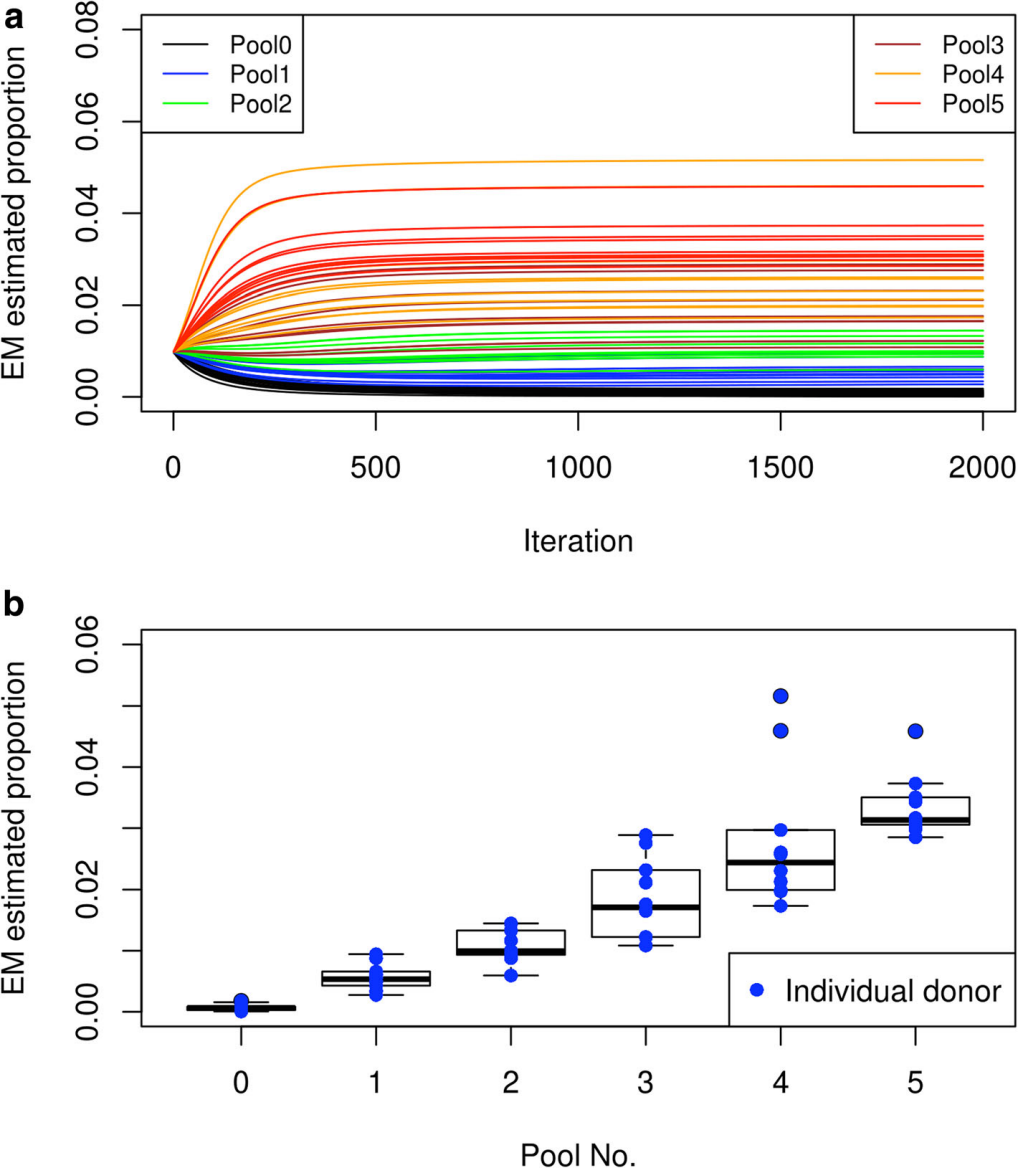
Estimating individual donor proportion of 102 PGP donors in a mixed pool. Individuals from Pool 0 are absent from the mixed pool while individuals from Pools 1, 2, 3, 4, and 5 are represented on average at 1X, 2X, 3X, 4X, and 5X, respectively (see “Methods”). **a** The estimated proportion (*y-axis*) at each iteration (*x-axis*) when running the algorithm. The different *colors* represent donors from different pools. **b** *Box plot* of the final estimate of the donor proportion for each pool. The *blue dots* indicate the estimate for each individual donor

To test if our method can accurately estimate the proportions of actual human donor samples, we set up a system using a pool of immortalized B-lymphocytes from the Harvard PGP [12–14]. We combined five pools of PGP B-lymphocytes with ten individuals per pool at 1X, 2X, 3X, 4X, and 5X concentration, respectively (see “Methods”). We extracted genomic DNA from the pool of B-lymphocytes and subjected the DNA to low-coverage whole-genome sequencing which resulted in the sequencing of 1,425,723 SNPs at 1.16X coverage. Using our method, we estimated the individual proportion of donors within the pool of 102 PGP individuals, including 52 donors that were not part of the combined pool and acted as negative controls. We found that the method predicted the proportion of the individuals within the pool (Fig. 5, Additional file 2: Table S5). The results showed that pool 0, which consists of the 52 individuals not part of the combined pool, had very low estimated proportions, with a mean proportion of 0.07% and none of the 52 samples had proportions > 0.18%. In contrast, pools 1–5 gave mean estimated proportions of 0.57%, 1.08%, 1.87%, 2.8%, and 3.35%, respectively, which accurately reflected the expected proportions (expected proportions being 0.67%, 1.33%, 2%, 2.67%, and 3.33%, respectively).

**Fig. 6:**
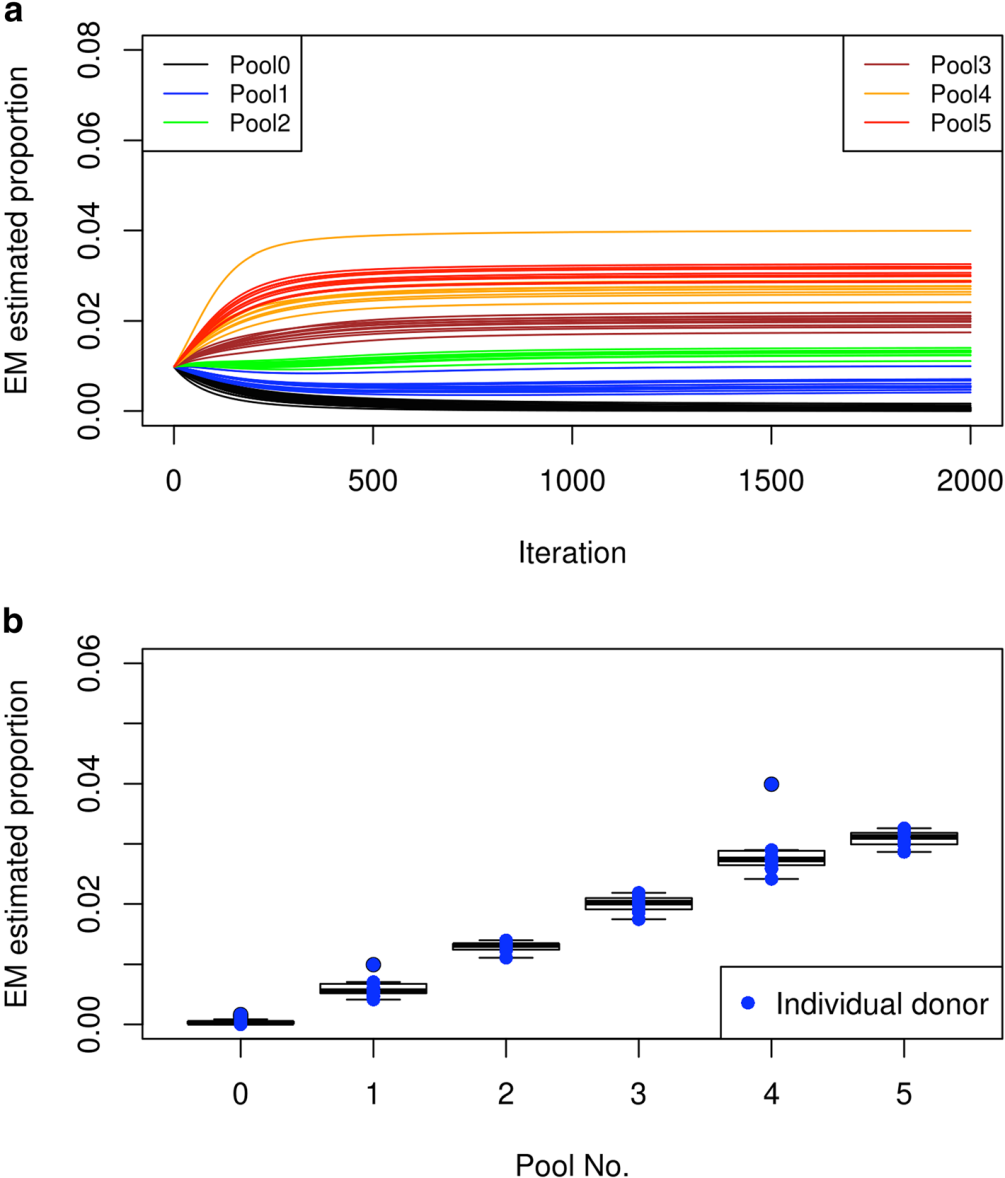
Estimating individual donor proportion after cell sorting each donor into a mixed pool. Similar to Fig. 5, but a cell sorter was used to accurately sort each individual donor cells into the mixed pool (see “Methods”). Note that the donors for each pool are different from those depicted in Fig. 5 (Additional file 2: Table S6). **a** The estimated proportion (*y-axis*) at each iteration (*x-axis*) when running the algorithm. The different *colors* represent donors from different pools. **b** *Box plot* of the final estimate of the donor proportion for each pool. The *blue dots* indicate the estimate for each individual donor

The initial pools had undergone a few rounds of passaging and they were created using a relatively inaccurate method for counting cells (Invitrogen Countess). Because of this, it is expected that the predicted individual proportions within each pool will vary greatly. We decided to repeat the experiment but with a more accurate way of determining the actual donor proportion before sequencing. Instead of using the pre-pooled cells, we chose a different set of 50 different donor cell lines to culture individually. We then sorted each donor cells using a cell sorter by assigning the number of live cell events (either 10,000, 20,000, 30,000, 40,000, or 50,000) for each donor to create the new pools (pools 1–5) (see “Methods”). Although there was a single outlier in pool 4 (hu52F345), we found that our method accurately predicted the proportion of the individuals within the pool (Fig. 6, Additional file 2: Table S6). The ranges of proportions for the different pools are as follows: pool 0 (0.00–0.16%); pool 1 (0.41–1%); pool 2 (1.11–1.4%); pool 3 (1.75–2.19%); pool 4 (2.41–3.99%); pool 5 (2.87–3.26%) (Additional file 2: Table S6). We observed that pools 0–5 gave mean estimated proportions of 0.03%, 0.6%, 1.29%, 1.99%, 2.84%, and 3.08%, respectively, which accurately reflected their actual proportions (expected proportions being 0%, 0.67%, 1.33%, 2%, 2.67%, and 3.33%). Taken together, our results demonstrate that our method can accurately predict the proportions of real samples where the donor genotypes are known through whole-genome sequencing or otherwise.

## Discussion

Various ways of pooling and sequencing DNA from multiple individuals in an effort to save costs in identifying genetic variants associated with disease status have been extensively investigated in genome-wide association studies [15]. Here, we propose a radically different use of whole-genome sequencing of pools of individuals: to enable the accurate prediction of individual donor proportion of a mixed pool of human tissue samples or cell lines. Human tissue samples and cell lines are the bedrock of biomedical research and their uses have been vital for many scientific discoveries. More recently, the development of induced pluripotent stem cells (iPSCs) derived from human tissue have allowed researchers to model a variety of cell types from any given patient [16–18]. Hence, technologies that improve our capability to perform high-throughput assays for phenotypes from cell lines will be increasingly more important, especially in the age of personalized medicine.

We described a method, which can accurately predict the individual donor proportion of a mixed pool of samples from many different donors without the need for artificial barcodes or amplification of a specific locus. Depending on the host and cell type, introducing artificial barcodes to every donor cell may not be practical or feasible to perform for large numbers of different donors. Also, PCR amplification of exogenous barcodes may potentially bias the results, as demonstrated by previous experiments when performed on mixtures of template DNA [19–22]. The use our method avoids the need to barcode every donor cell or PCR amplification of a specific locus.

As our method effectively uses many SNPs present in the host genome as input to identify donor proportion, it is not suitable for applications where such SNPs are not present. For example, previous research reported the use of 20-nt barcodes to simultaneously create and tag a library of yeast deletion mutants using mitotic recombination for high-throughput multiplex assays [23, 24]. The library of deletion mutants was created from cells from a single donor and our method would not be able to differentiate between different deletion mutants as their genome-wide SNP profile are identical. On the other hand, when multiple donor cells are used like the study that interrogated multiple cancer cell lines from different donors [7], our method would be highly effective for identifying the proportion of different donor cells without the need for DNA barcodes. Our method can also be adjusted for parallel model organism screens, i.e. pooling of cells from different organisms to be interrogated together. If the genomes of the organisms are different enough, the problem becomes trivial, as it is possible to determine the origin of each of the sequencing reads by alignment to the right host genome. However, if the genomes of the various model organisms were similar, the main genetic difference between them may not be SNPs but other polymorphisms such as insertion-deletion polymorphisms. We can incorporate these polymorphisms or other types of genetic variants into our method for such use.

Experimentally, all that is required is genomic DNA extraction and whole-genome sequencing of the extracted DNA. The prediction of individual donor proportion is then determined computationally. Our described method enables the multiplexing of phenotypic assays on multiple different donor samples in a single experiment, which significantly reduces effort and time and facilitate discoveries. Our method can be used for high-throughput measurements of various cellular phenotypes for the purpose of discovering genetic alleles associated with cellular phenotypes, similar to those performed on human traits and diseases obtained from medical record data [25–30]. While there are substantially fewer such studies of cellular phenotypes, we predict that our method would greatly accelerate such discoveries of cellular phenotypes by facilitating and enabling researchers to perform multiplexed testing of diverse donor cells *en masse*. Whether the cells are sorted via FACS or selected for via different growth conditions, the resulting proportion for each donor within the sorted or selected pool can be accurately estimated using our method, resulting in the simultaneous testing of numerous different donor cells in a single experiment (Fig. 1). Current work in our laboratory is focused on utilizing this method to perform multiple phenotype characterization on thousands of cell lines from PGP and other cohorts to uncover genetic alleles associated with these phenotypes. We have also made the software for estimating donor proportion as well as performing the simulation experiments freely available (see “Availability of data and material”) so that other groups can harness our method for their research experiments as well

## Conclusions

In summary, we have developed a method to accurately predict the individual proportion from a mixed pool of cells from different donors without artificial barcodes or amplification of a specific locus. The method enables the simultaneous testing of cells from a pool of different donors and is transformative for scaling up the number of donor samples used. Instead of performing lentiviral barcoding manually for each donor sample, our method relies on having whole-genome genotype information for each donor, which is now readily available for many samples. Our method lowers the costs and associated resources for performing such experiments and would help facilitate multiplexed experimentation on large cohorts of donor cells.

## Additional files

Additional file 1: **Note S1–2.** working example of the method and comparison of the method against PRISM. (PDF 278 kb)

Additional file 2 **Table S1–6.** all supplementary tables. (XLSX 638 kb)

## Abbreviations

EM: Expectation-maximization
FACS: Fluorescence-activated cell sorting
MAF: Minor allele frequency
PGP: Personal Genome Project
SNP: Single nucleotide polymorphism
