# Supplementary material for: Enabling multiplexed testing of pooled donor cells through whole-genome sequencing
Source: Genome Med. 2018 Apr 19;10:31. doi: 10.1186/s13073-018-0541-6 (PMC5909281; doi:10.1186/s13073-018-0541-6)
Supplement: Supplementary file 1 — Note S1–2. working example of the method and comparison of the method against PRISM. (PDF 278 kb) [file 13073_2018_541_MOESM1_ESM.pdf]

# Note S1

## A working example of the application of the method

To illustrate the method, we will go through a working example of a mixed pool of 5 distinct donors using 10 SNPs and a read-depth (coverage) of 100. We first assume the individual proportion of the 5 donors to be 0, 0.1, 0.2, 0.3 and 0.4 respectively, the sum of which adds up to 1 (See table below).

| | $Donor_1$ | $Donor_2$ | $Donor_3$ | $Donor_4$ | $Donor_5$ |
|---|---|---|---|---|---|
| Proportion | 0.0 | 0.1 | 0.2 | 0.3 | 0.4 |

Next, we generate genotypes for 10 SNPs for each donor, where each SNP is bi-allelic with either the reference allele (R) or the alternate allele (A) and the possible genotypes are R/R, R/A or A/A denoting RR homozygous, RA heterozygous or AA homozygous genotypes. The generated genotypes are shown in the table below.

| SNP | $Donor_1$ | $Donor_2$ | $Donor_3$ | $Donor_4$ | $Donor_5$ |
|---|---|---|---|---|---|
| 1 | R/R | A/A | R/R | R/R | R/R |
| 2 | R/A | A/A | R/A | A/A | A/A |
| 3 | R/A | R/R | A/A | A/A | R/R |
| 4 | R/R | A/A | R/A | A/A | A/A |
| 5 | R/R | R/A | R/A | R/R | R/R |
| 6 | R/R | R/A | R/R | R/R | R/A |
| 7 | R/A | R/R | R/R | R/A | R/R |
| 8 | A/A | R/A | A/A | R/A | R/R |
| 9 | R/R | R/R | R/R | R/R | R/A |
| 10 | R/R | R/A | R/A | A/A | R/R |

Next, we generate sequencing reads covering the SNPs and count the number of read representing the R and A allele. If the sequencing depth/sampling is high enough, the number of R and A allele count will reflect the expected allele fraction given the individual donor proportion and the genotypes (See table below).

| SNP | Expected R allele fraction | R allele count | A allele count |
|---|---|---|---|
| 1 | 0.9 | 90 | 10 |
| 2 | 0.1 | 10 | 90 |
| 3 | 0.5 | 50 | 50 |
| 4 | 0.1 | 10 | 90 |
| 5 | 0.85 | 85 | 15 |
| 6 | 0.75 | 75 | 25 |
| 7 | 0.85 | 85 | 15 |
| 8 | 0.6 | 60 | 40 |
| 9 | 0.8 | 80 | 20 |
| 10 | 0.55 | 55 | 45 |

The R allele fraction for each SNP is calculated by taking the sum of the donor proportion multiplied by the probability of observing the R allele for that SNP. The probably for observing the R allele is 1, 0.5 and

0 for the genotypes R/R, R/A and A/A respectively. As such, for $SNP_1$, the R allele fraction is (0.0)(1) + (0.1)(0) + (0.2)(1) + (0.3)(1) + (0.4)(1), which is equal to 0.9. For $SNP_2$, the R allele fraction is (0.0)(0.5) + (0.1)(0) + (0.2)(0.5) + (0.3)(0) + (0.4)(0), which is equal to 0.1. Also, in this working example, we are assuming that the observed R and A allele count matches the expected R allele fraction, but when we perform the simulations as described in the main section, the R and A allele count were sampled from a binomial distribution with the expected R allele fraction as the parameter.

With these parameters in place, we now apply our method to determine the individual donor proportion, which is an iterative process. At iteration 0 ($t$=0), we assume that the individual donor proportions are equal, i.e. 1/5 which is 0.2 (See table below).

| Iteration ($t$) | Estimated $Donor_1$ | Estimated $Donor_2$ | Estimated $Donor_3$ | Estimated $Donor_4$ | Estimated $Donor_5$ |
|---|---|---|---|---|---|
| 0 | 0.2 | 0.2 | 0.2 | 0.2 | 0.2 |

We next calculate Total R and Total A for each SNP by using the formula given in the Methods section of the manuscript (See table below).

| SNP | Total R | Total A |
|---|---|---|
| 1 | 0.8 | 0.2 |
| 2 | 0.2 | 0.8 |
| 3 | 0.5 | 0.5 |
| 4 | 0.3 | 0.7 |
| 5 | 0.8 | 0.2 |
| 6 | 0.8 | 0.2 |
| 7 | 0.8 | 0.2 |
| 8 | 0.4 | 0.6 |
| 9 | 0.9 | 0.1 |
| 10 | 0.6 | 0.4 |

Total R is calculated by taking the sum of the current estimated donor proportion (in this case it is 0.2 but will change with each iteration of the algorithm) multiplied by the probability of observing the R allele for that SNP. The probably for observing the R allele is 1, 0.5 and 0 for the genotypes R/R, R/A and A/A respectively. As such, for $SNP_1$, the Total R is (0.2)(1) + (0.2)(0) + (0.2)(1) + (0.2)(1) + (0.2)(1) = 0.8. The Total A for the same SNP is just 1 – Total R, i.e. for $SNP_1$, the Total A is 1 – 0.8 = 0.2. For $SNP_2$, the Total R is (0.2)(0.5) + (0.2)(0) + (0.2)(0.5) + (0.2)(0) + (0.2)(0) = 0.2 and the Total A is thus 0.8. The Total R and Total A for the rest of the SNPs can be similarly calculated.

Next, the likelihood function $L$ for each individual is calculated using the formula given in the Methods section of the manuscript (See table below).

| $L$ $Donor_1$ | $L$ $Donor_2$ | $L$ $Donor_3$ | $L$ $Donor_4$ | $L$ $Donor_5$ |
|---|---|---|---|---|
| 172.986 | 199.325 | 184.593 | 210.784 | 232.311 |

The $L$ of $Donor_1$ is calculated by the sum of the fractional proportion of the observed reads given the genotypes for each SNP (See table below).

| SNP | Genotype $Donor_1$ | Calculation | Result |
|---|---|---|---|
| 1 | R/R | (0.2/0.8)*90 | 22.5 |
| 2 | R/A | 0.5*((0.2/0.2)*10 + (0.2/0.8)*90) | 16.25 |
| 3 | R/A | 0.5*((0.2/0.5)*50 + (0.2/0.5)*50) | 20 |
| 4 | R/R | (0.2/0.3)*10 | 6.667 |
| 5 | R/R | (0.2/0.8)*85 | 21.25 |
| 6 | R/R | (0.2/0.8)*75 | 18.75 |
| 7 | R/A | 0.5*((0.2/0.8)*85 + (0.2/0.2)*15) | 18.125 |
| 8 | A/A | (0.2/0.6)*40 | 13.333 |
| 9 | R/R | (0.2/0.9)*80 | 17.778 |
| 10 | R/R | (0.2/0.6)*55 | 18.333 |

The sum of the Result column adds up to 172.986, which matches the $L$ for $Donor_1$. The $L$ for the other donors can be similarly calculated.

Finally, the new estimated donor proportion ($t$=1) is calculated by taking the individual donor likelihood divided by the sum of all the likelihoods. The sum of all the likelihoods is 172.986 + 199.325 + 184.593 + 210.784 + 232.311 = 1000. The new estimated donor proportion is (See table below),

| $t$ | Estimated $Donor_1$ | Estimated $Donor_2$ | Estimated $Donor_3$ | Estimated $Donor_4$ | Estimated $Donor_5$ |
|---|---|---|---|---|---|
| 1 | 0.172986 | 0.199325 | 0.184593 | 0.210784 | 0.232311 |

The procedure is now repeated with a new iteration and the new estimated donor proportion is used to calculate the next estimated donor proportion. The results for the next 30 iterations are as follows (See table below),

| $t$ | Estimated $Donor_1$ | Estimated $Donor_2$ | Estimated $Donor_3$ | Estimated $Donor_4$ | Estimated $Donor_5$ |
|---|---|---|---|---|---|
| 2 | 0.152089789 | 0.194923147 | 0.172601406 | 0.220723518 | 0.25966214 |
| 3 | 0.135620518 | 0.188587685 | 0.163310245 | 0.230108468 | 0.282373084 |
| 4 | 0.122415496 | 0.181460242 | 0.156136279 | 0.239020444 | 0.300967539 |
| 5 | 0.111660358 | 0.174229097 | 0.150622851 | 0.247443578 | 0.316044116 |
| 6 | 0.102774384 | 0.16728513 | 0.146418224 | 0.25532604 | 0.328196222 |
| 7 | 0.095336301 | 0.160831154 | 0.143252531 | 0.26261378 | 0.337966234 |
| 8 | 0.089035491 | 0.154954744 | 0.140918156 | 0.269267185 | 0.345824425 |
| 9 | 0.083639355 | 0.149675181 | 0.139254243 | 0.275267286 | 0.352163934 |
| 10 | 0.078971151 | 0.144973057 | 0.138134919 | 0.28061591 | 0.357304962 |
| 11 | 0.074894729 | 0.140808603 | 0.137460516 | 0.285332696 | 0.361503456 |
| 12 | 0.071303889 | 0.137132838 | 0.137151112 | 0.289450863 | 0.364961299 |
| 13 | 0.068114856 | 0.133894194 | 0.137141822 | 0.293012845 | 0.367836283 |
| 14 | 0.065260882 | 0.131042307 | 0.137379356 | 0.296066414 | 0.370251042 |
| 15 | 0.062688324 | 0.128530051 | 0.137819504 | 0.298661521 | 0.3723006 |
| 16 | 0.060353743 | 0.126314513 | 0.138425281 | 0.300847914 | 0.374058549 |
| 17 | 0.058221729 | 0.12435731 | 0.139165541 | 0.302673461 | 0.375581959 |

| | | | | | |
|---|---|---|---|---|---|
| 18 | 0.056263252 | 0.122624538 | 0.140013932 | 0.30418306 | 0.376915218 |
| 19 | 0.054454388 | 0.121086517 | 0.140948088 | 0.305418006 | 0.378093 |
| 20 | 0.052775329 | 0.119717426 | 0.141948991 | 0.306415715 | 0.379142539 |
| 21 | 0.0512096 | 0.118494907 | 0.143000468 | 0.307209674 | 0.380085352 |
| 22 | 0.04974344 | 0.11739966 | 0.144088774 | 0.307829568 | 0.380938558 |
| 23 | 0.048365311 | 0.116415065 | 0.14520225 | 0.308301501 | 0.381715873 |
| 24 | 0.047065493 | 0.11552683 | 0.146331036 | 0.30864828 | 0.382428361 |
| 25 | 0.045835764 | 0.114722674 | 0.147466824 | 0.30888972 | 0.383085018 |
| 26 | 0.044669142 | 0.113992044 | 0.148602649 | 0.309042958 | 0.383693206 |
| 27 | 0.043559663 | 0.113325878 | 0.149732708 | 0.309122755 | 0.384258996 |
| 28 | 0.042502215 | 0.112716382 | 0.150852199 | 0.309141778 | 0.384787425 |
| 29 | 0.04149239 | 0.112156853 | 0.15195719 | 0.309110862 | 0.385282704 |
| 30 | 0.04052637 | 0.111641515 | 0.153044494 | 0.309039248 | 0.385748374 |

The estimated donor proportion approaches the actual proportion with each iteration of the algorithm. When the number of iteration reaches *t*=100, the estimated proportion closely matches the actual proportion and when t=2000, the estimated proportion more accurately matches the actual proportions of 0, 0.1, 0.2, 0.3 and 0.4 (See table below).

| *t* | Estimated $Donor_1$ | Estimated $Donor_2$ | Estimated $Donor_3$ | Estimated $Donor_4$ | Estimated $Donor_5$ |
|---|---|---|---|---|---|
| 100 | 0.01399887 | 0.10252427 | 0.185392924 | 0.301948963 | 0.39613499 |
| 2000 | 6.32E-04 | 0.100094786 | 0.199373627 | 0.300057613 | 0.39984154 |

To illustrate the algorithm better, we show a graph of how the estimated donor proportion changes with each iteration (See figure below).

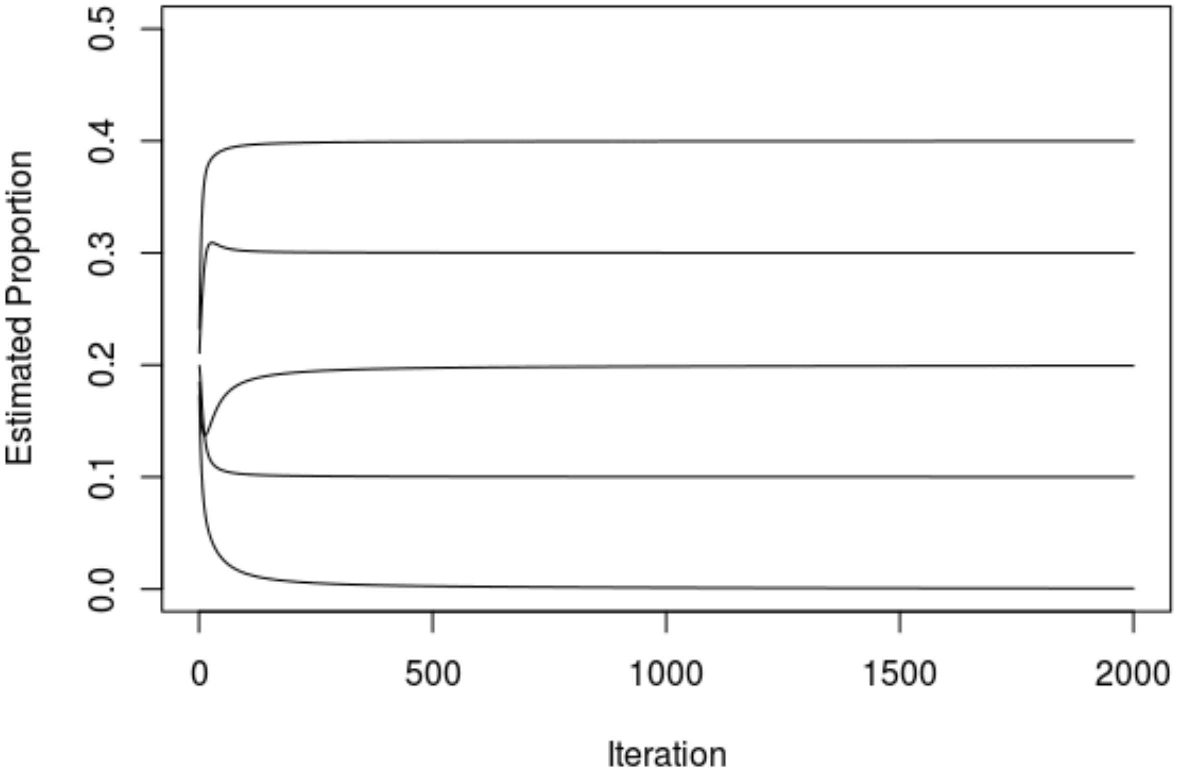

# Note S2

## Comparing our proposed method against the PRISM method

Method using lentiviral barcodes (PRISM)

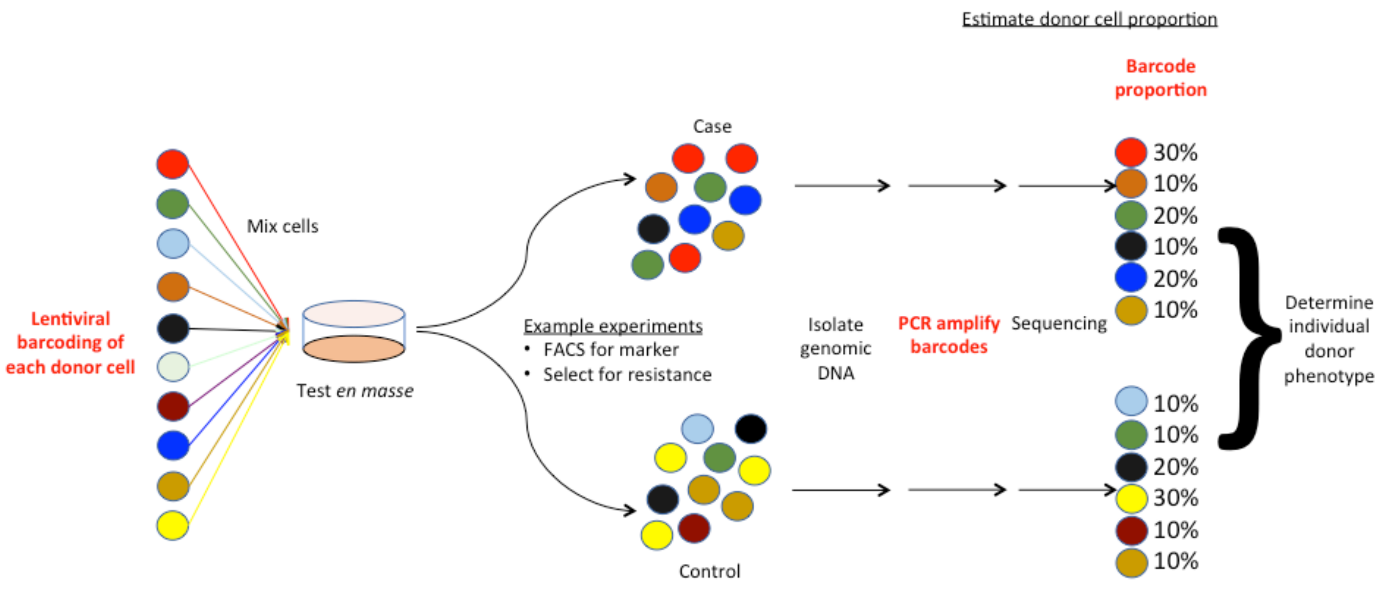


Our Method

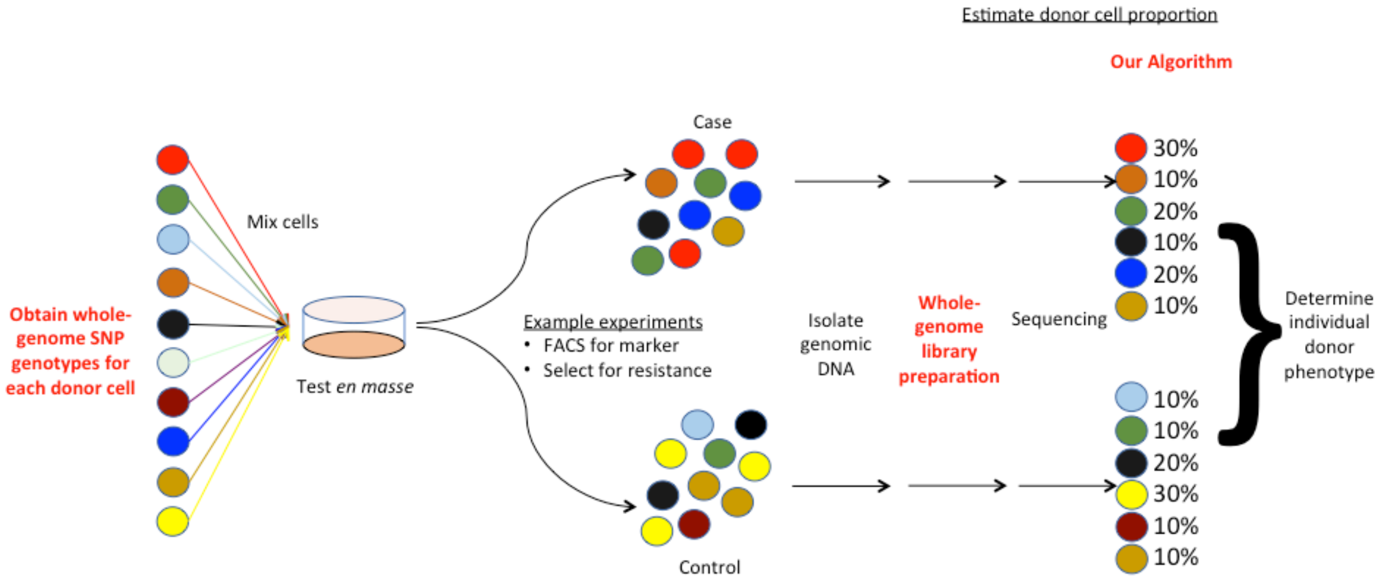


The above figure illustrates the difference between our method and the lentiviral barcode method described in Yu et. al. (PRISM) [7]. The steps that differ between the 2 methods are listed in red. For the PRISM method, each donor cell must be uniquely barcoded. This can be done by individually infecting each donor cell line with a specific lentivirus to integrate a unique barcode for each donor. Individual clones for each donor are then selected for successful barcode integration. The donor cells with integrated barcodes are then mixed together for further downstream experiments. The use of our method avoids the need to introduce exogenous barcodes into the donor cells. Instead, our method requires whole-genome SNP genotypes for each donor. This can be achieved by downloading whole-genome sequencing data available for each donor. If such data is not already available, DNA from each

donor obtained from the cells or from the donor directly can be sent for whole-genome SNP genotyping using SNP arrays or whole-genome sequencing. In either case, the mixed donor cells are then subjected to a phenotypic assay, where cells are sorted or selected into Case and Control groups. For each of the groups, genomic DNA is extracted and isolated. For the PRISM method, fixed primers flanking the barcode locus are used to PCR amplify the barcodes. For our method, we instead perform whole-genome library preparation (e.g. Nextera DNA Library Preparation by Illumina), just like how one would do for preparing DNA for whole-genome sequencing. The prepared samples are then sent for next-generation sequencing. For the PRISM method, the proportion of donor cells in each group can be read out directly from the sequencing results by calculating the proportion of sequencing reads that contain the specific barcode used to tag the specific donor. For our method, the proportion of donor cells is calculated using our method's algorithm, which employs an Expectation-Maximization procedure to estimate each donor's proportion by comparing the sequencing read with the known whole-genome SNP profile for every individual donor. Regardless of the method used, each individual donor phenotype can then be determined by comparing the difference between each individual donor proportion between the Case and Control groups. The Table below highlights the differences between the PRISM method and our method.

| PRISM method | Our Method |
|---|---|
| Requires introduction of unique DNA barcodes into each donor cell via lentiviral integration. | Requires whole-genome SNP genotypes for each donor via SNP-arrays or otherwise. |
| PCR amplification of barcodes | Whole-genome library preparation |
| Donor proportion determined by proportion of barcodes that identify the donor from the sequencing reads. | Donor proportion determined by our algorithm, which employs an Expectation-Maximization procedure to compare the sequencing reads with each donor whole-genome SNP profile. |
